# Supplementary material for: “Climate Change and Health?”: Knowledge and Perceptions among Key Stakeholders in Puducherry, India
Source: Int J Environ Res Public Health. 2023 Mar 7;20(6):4703. doi: 10.3390/ijerph20064703 (PMC10048771; doi:10.3390/ijerph20064703)
Supplement: Supplementary file 1 [file ijerph-20-04703-s001.zip › ijerph-2204573-supplementary.pdf]

Supplementary material

1. Interview questions guide for medical professionals and members of the Department of Science, Technology and Environment (DSTE) working on the Puducherry State Adaptation plan

COMMON QUESTIONS

Target group	Themes	Aims	Questions
COMMON	Participant information	To get the basic demographic and professional information about the participant	1. Age<br>2. Nationality<br>3. Place of residence<br>4. Educational background and speciality<br>5. Occupation<br>6. Years in occupation<br>7. Professional experience related to climate change and/or health
	Background knowledge-climate change	To establish level of knowledge about climate change with 'warm up' questions	1. What comes to mind when you think of climate change?<br>2. What do you think are physical manifestations of climate change?<br>3. Have you experienced or been aware of any climate change events in the past few years?<br>4. What, according you, are the most common consequences of climate change?- Which aspects of life do they affect most severely?
	Climate change and health, climate change and CVDs	To establish knowledge/ awareness about climate change and health and climate change and CVDs	1. Have you ever thought about the health impacts of climate change?<br>2. What aspects of human health do you think climate change will have the biggest impact on?<br>3. Are you aware about the impacts of climate change on NCDs such as cardiovascular diseases?<br>3.1. If yes to above question, what do you know about it? How do you know about it (eg through

			research or through professional experience?)
	Policies and plans	To establish knowledge and awareness levels about policies and plans on the issue of climate change and health	1. Are you aware or have you been part of any policies/plans/programs on the issue of climate change and health?<br>2. If yes, what were/are they?- What diseases or health topic did it focus on? What was the work done (aim)? Do you think it was successful and beneficial?<br>3. Do you know any plans/programs specifically targeting climate change and heart diseases? (can be from any sector).<br>4. Do you know about the national/state climate change adaptation plan?<br>5. If yes, do you know of the role health plays in it?
	Challenges and outlook	To understand challenged faced, potential solutions and planned changes	1. Are you aware of any upcoming or recent changes to the health adaptation plans or any other relevant policy that target climate change and health?<br>2. Do you know any policies which can be used to increase awareness and research on the impact of climate change on CVDs in India?<br>3. According to you, what are the biggest drawbacks and challenges faced- why do you think health or NCD impacts of climate change are not a priority?<br>4. What can be done to change that?<br>5. Can you think of some measures to mitigate the impacts of climate change?<br>- Your contribution to mitigating the impact, whether it's individual or you think it should be

			more at a governmental level?<br>- Examples can be green healthcare facilities etc
Medical Professionals	Climate change and health-medical experience	To understand perceptions on the extent to which climate/temperature affects patient health	1. Do you think climate, especially temperature affects health based on hospital admissions and mortality?<br>2. Can you explain what you have observed (eg, more patients on particularly hot days).<br>3. Which diseases have you observed to be the most sensitive to climate/temperature?<br>4. Based on your day to day observations, do you see an association between temperature and CVDs?<br>5. Do you think we will see an increase in the CVD deaths attributable to temperature in the future?
	Population vulnerability	To understand views on how different people are affected based on demography	1. Which people have you observed to be the most vulnerable to temperature? (eg, age, gender, occupation, SE status etc)<br>2. Do you see a big gender difference in CVD patients with and without the influence on external temperatures?<br>3. Have you observed an association between age/gender and temperatures? For example, are a certain group of people more susceptible to heat or cold?<br>4. Do the public and private sectors work together during disasters?<br>- How is the communication, facility and equipment sharing?
	Education and training	To understand level of training and awareness among	1. Have you ever been explicitly trained, either during medical

		doctors on climate sensitive diseases	school or professionally, on climate sensitive diseases?<br>2. If yes, where did the course take place (India or abroad, college or professional) and what did it cover (broadly)<br>3. If yes, did the course include CVDs?<br>4. Did it include gender differences in terms of symptoms?<br>5. Do you think such a course is needed or would be beneficial?
	Measures to be taken	To discuss possible measures to be taken to increase awareness and preparedness on climate sensitive diseases	1. Assuming that we will be seeing an increase in CVD mortalities attributable to temperature in the future, what measures do you think can be taken to prepare and adapt to it?<br>- In hospitals, in medical schools,<br>- Give an example..early warning systems, emergency cardio bays in hospitals, awareness drives etc<br>2. What policy measures do you feel would benefit with the issue of climate sensitive diseases? For CVDs?
Health department (policy makers, ministerial representatives)	Current policies	To understand current health policies and whether climate change in included in them- include CVD policies and training	1. Do any of the current health policies include climate change?<br>2. Are there any specific policies on climate sensitive diseases?<br>- Guidelines for disasters<br>- Guidelines for heat<br>3. If yes, do any policies include NCDs or CVDs specifically?
	Challenges and outlook	To understand current challenges and future plans	1. The national adaptation plan recently added health as one of its climate change missions- why do you think this was not always a priority?<br>2. FOR PUDUCHERRY- Why is there no health mission in the state adaptation plan? Are

			there plans to include it? If yes, what diseases will be focused on?<br>3. Are any activities being planned around climate sensitive diseases especially CVDs?<br>- Awareness programs<br>- Education and training in medical schools?<br>4. Are there any plans to develop and expand heat action plans nationally? (eg. Ahmedabad heat action plan)<br>5. Which diseases will be a priority area for climate sensitivity in terms of policy and research?<br>6. Is there any research or study being conducted on the health impacts of climate change? Are there any plans to do so?<br>7. CVD effects and most other effects of climate change affect vulnerable populations most-how do you plan to address some of these challenges?<br>- Rural vs urban exposures and requirements differ. Are there plans that specifically target different populations?<br>- What about different communities? Do you feel different communities have different needs to protect themselves against the climate? Can you elaborate?<br>- What about different SE groups?
Department of Science, Technology and Environment	Current policies	To understand the development process of the adaptation plan and how health is included in current policies	1. The adaptation plan has recently added a health mission, but it does not have a comprehensive list and guidelines for climate sensitive disease management?

			2. The adaptation plan is an intersectional plan with involvement from many departments. Does the steering committee have regular meetings to review and stay perpared, if so how often?<br>- Review meetings?<br>- Do you make changes to the plan based on new evidence or incidences?<br>3. Is there a reason that NCDs such as CVDs, despite having a huge burden of disease are not explicitly included and researched in terms of climate attributable burden?<br>4. What plans/programs/campaigns are presently ongoing that deal with climate-health adaptation? Are there any specific to CVDs?<br>5. Can you tell me about any plans or policies that have been previously implemented on the topic of climate sensitive diseases?<br>- Were they successful and effective?<br>- What is the present status of these?<br>- What were the biggest challenges faced?<br>6. CVD effects and most other effects of climate change affect vulnerable populations most-how do you plan to address some of these challenges?<br>- Rural vs urban exposures and requirements differ. Are there plans that specifically target different populations?<br>- What about different communities? Do you feel different communities have

			different needs to protect themselves against the climate? Can you elaborate?<br>- What about different SE groups?
	Challenges and outlook	To understand challenges faced and future plans	1. What is the biggest challenge in formulating and implementing climate sensitive disease policies or policies related to climate impacts on health?<br>2. Are there any plans to include NCDs and CVDs as a separate component of climate sensitive diseases?<br>3. Are there research projects planned on this topic to inform priority setting and policy formulation?<br>4. What measures do you think need to be taken to help formulate better , more comprehensive climate change policies that are informed by health research?
			The adaptation plan is an intersectional plan with involvement from many departments. Does the steering committee have regular meetings to review and stay perpared, if so how often?<br>1. Review meetings?<br>2. Do you make changes to the plan based on new evidence or incidences?<br>3.
EDUCATION- either department or medical college educators	Current practices	To understand current medical curriculum and whether climate change is included in it	4. Does the current medical school curriculum include climate change with respect to diseases that are sensitive to it?<br>5. Do you feel this needs to be taught?<br>6. Do you think temperature affects diseases?<br>7. What diseases do you think are most sensitive to climate change that need to be included in the curriculum?

			8. If yes to above questions, does it include gender differences?
	Challenges and outlook	To understand challenges in teaching students about climate change and plans for future along with suggestions	1. Are there any plans to include climate sensitive diseases in the curriculum?<br>2. What other measures do you think can be taken with respect to the training of medical workers to reduce the burden of climate sensitive diseases?<br>- Ex, emergency bays, more ambulances or staff during days of particular temperature etc

Table S1: Interview guideline for key-informant interviews

2. Themes and categories

Theme	Sub-theme	Category
Climate change and health: systems knowledge and perceptions	Climate change process	1. Climate change as an acute and growing problem for India<br>2. Climate change ultimately affects health through domino effects<br>3. Indirect health impacts of climate change experienced<br>4. Scepticism about climate change affecting CVDs<br>5. Knowledge of the public health burden and vulnerabilities influenced perceived risks from climate change
	Climate change impacts	
	Risk factors	
	Disease burden	
	Vulnerabilities	
Socio-cultural dynamics and public engagement	Awareness and outreach	1. Credibility and societal role of information source important for uptake and public awareness<br>2. Need for alternate solutions and incentivized, targeted programs on all societal levels<br>3. Integrated climate change impacts in schools, universities and continuing education curriculums<br>4. Seasonal workplace guidelines
	Capacity strengthening	
	Adaptive action	
Institutional determinants	Policy support	
	Education	
	Research and challenges	

Table S2: Structural codes and framework matrix

3. Additional supporting quotes

   a. Systems knowledge: Climate change is an acute, growing problem for India

      "…I think nowadays, we cannot predict when the rains are coming and when it is going to go. I think more of cyclones are coming….we will have a drought and the other year, we will have a flood. That's what is happening nowadays" #2, Medical doctor/researcher.

   b. Systems knowledge: Indirect impacts of climate change experienced

      "Yeah it affects agriculture. It makes people unemployed. The more agriculture commodities that get damaged, so we cannot do anything about the agricultural damage." #1, Practicing physician.

      "Water is one of the important sectors in which I feel that climate change will have a larger impact. Because already due to the overpopulation, there's a stress in the groundwater resources. And because of the climate change, the increasing heat, the soil moisture will get affected and there will be a lot of stress on the aquifers also. As the surface water also gets-evaporates due to increase in temperature, I see the possibilities of the future where there will be a lot of competition for the water." #7, Environmentalist.

   c. Systems knowledge: Skepticism about climate change affecting CVDs

      "I work in cardiology department; we didn't feel like there is increased deaths or increased number of myocardial infarction during the season in the period." #12, Practicing physician.

   d. Socio-cultural dynamics and public engagement: Credibility and societal role of information source is important for uptake and public awareness

      "Once we have accumulated the knowledge about this disease and the climate, we can make a manual or we can issue it to the patients with the support of the newspaper, radio and television so you can make…We can advise about the preventive measures." #13, Practicing physician.

   e. Socio-cultural dynamics and public engagement: integrating climate change in schools, universities and continuing education curriculum

      "And especially as a teacher, I would like this to be a part of the curriculum also. Because we don't have a separate chapter on climate change or you know. I think, more of, expanding the curriculum from school to the collegiate also. Because ultimately, COVID has (taught us) that, you know, many things are not always under our control. So climate change is another one." #2, Medical doctor/researcher.

      "Medical UG courses itself it should be added in. Input of what is climatic change what we are going to face and how it's a disaster and how to do the system management…First we

need to teach to doctor students, then they'll be able to teach to others actually." #6, Practicing physician.
